# Prognostic impact of tumour–vessel proximity in soft tissue sarcoma: Fujiwara classification provides enhanced risk stratification

**DOI:** 10.1186/s12957-026-04354-y

**Published:** 2026-04-16

**Authors:** Christian Spiegel, Lara Huettl, Friederike Weschenfelder, Karin Gabriela Schrenk, Thomas Ernst, Wolfram Weschenfelder

**Affiliations:** 1 Department of Trauma, Hand and Reconstructive Surgery and Orthopaedics, University Hospital Jena, Am Klinikum 1, Jena, 07747 Germany; 2 Comprehensive Cancer Center Central Germany, Campus Jena, Jena, Germany; 3 Department of Obstetrics, University Hospital Jena, Jena, Germany; 4 Department of Haematology and Internal Oncology, Clinic of Internal Medicine II, University Hospital Jena, 07747 Jena, Germany

## Abstract

**Background:**

Tumour proximity to major neurovascular structures complicates resection of deep soft tissue sarcomas (STS), yet its independent impact on overall survival (OS) remains unclear. This study assessed whether anatomical proximity, measured by dichotomised distance and the Fujiwara classification, predicts oncologic outcomes after curative-intent surgery.

**Methods:**

Patients with high-grade extremity or truncal STS treated between 2004 and 2020 were retrospectively analysed. Minimal tumour–vessel distance (> 1 cm vs. ≤ 1 cm) and Fujiwara type (I–IV) were determined from preoperative MRI. OS was evaluated using Kaplan–Meier and Cox regression. Multivariable Cox models were pre-specified to include established prognostic confounders (age, tumour size, histologic grade, and resection status), irrespective of univariate significance. Fujiwara types III and IV were pooled to improve estimate stability.

**Results:**

A total of 113 patients met inclusion criteria (median follow-up 84 months); 47 deaths occurred during follow-up. Tumour proximity ≤ 1 cm was associated with significantly reduced OS (42 vs. 107 months; *p* = 0.003). The Fujiwara classification was associated with significant survival differences across categories (*p* < 0.001).

In multivariable analysis (Model A), proximity ≤1 cm remained independently associated with OS (HR 3.04; 95% CI 1.38–6.67; p = 0.006), together with age ≥65 years (HR 3.42; p = 0.007) and R2 resection (HR 9.67; p = 0.002). In Model B, pooled Fujiwara type III/IV remained independently associated with impaired OS (HR 3.62; 95% CI 1.47–8.94; p = 0.005), alongside age ≥65 years (HR 3.07; p = 0.015) and R2 resection (HR 7.32; p = 0.007). Higher Fujiwara types correlated with increased local recurrence (p = 0.011), while distant metastasis rates were similar across groups.

**Conclusions:**

Tumour proximity to neurovascular structures is independently associated with OS in high-grade deep STS. The Fujiwara classification remained prognostically relevant after adjustment for established risk factors including resection status. These findings suggest that anatomical tumour–vessel relationships provide complementary prognostic information beyond conventional clinicopathologic variables. Prospective multicentre validation is warranted.

## Introduction

Soft tissue sarcomas (STS) comprise a heterogeneous group of malignant mesenchymal neoplasms that together account for approximately 1% of adult cancers [1, 2]. The current WHO classification recognises more than 100 histologic subtypes with distinct biological behaviours and therapeutic implications [3]. Incidence varies regionally between about 1.8 and 5.1 per 100,000 persons per year and increases with age, peaking in the sixth decade of life [1, 4, 5]. Management requires specialist multidisciplinary care because local resectability, the feasibility of oncologic margins and metastatic potential differ markedly between entities [2, 3, 5].

Established prognostic determinants include tumour size, depth, histologic grade (FNCLCC) and resection status, with R0 resection being the strongest predictor of durable local control [6–9]. Model-based tools such as Sarculator and PERSARC improve individualised survival estimates by integrating clinical and pathologic variables, but do not explicitly incorporate anatomical relationships to major neurovascular structures [10–17].

The Fujiwara classification is an MRI-based system that quantifies tumour–vessel relationships by assigning four types according to minimal distance and circumferential vessel contact (> 5 mm to ≥ 180°) [18]. Originally developed for femoral osteosarcoma, it has been extended to deep soft tissue tumours and has been associated with stepwise increases in local recurrence and greater surgical complexity [19, 20]. Prior reports indicate that even when vessel resection and reconstruction are performed, higher Fujiwara types remain associated with elevated local recurrence risk, suggesting that the prognostic signal may derive from the broader neurovascular compartment and surgical constraints rather than from the vessel itself [19, 21].

Given limited and heterogeneous evidence for the prognostic significance of tumour–vessel proximity in STS, we hypothesised that closer tumour proximity to major neurovascular structures adversely affects oncologic outcomes following curative resection. We therefore analysed the impact of both a simple dichotomised proximity measure (> 1 cm vs. ≤ 1 cm) and the Fujiwara classification on overall survival (OS), and explored potential mechanisms—in particular local recurrence—underpinning observed associations while adjusting for established prognostic variables.

## Materials and methods

### Study population

This retrospective study included patients from the institutional sarcoma registry who underwent surgical treatment for extremity or truncal soft tissue sarcomas between 2004 and 2020. Intra-abdominal and retroperitoneal tumours were excluded because the Fujiwara classification is not applicable to these anatomical compartments. The objective of the study was to evaluate oncologic outcomes and prognostic factors in patients treated with curative intent. Of 216 patients initially identified, those with low-grade sarcomas (G1) were excluded (*n* = 49) to focus the analysis on high-grade disease. An additional 9 patients were excluded due to missing baseline imaging, and 11 patients due to incomplete follow-up data. To allow assessment of prognosis after curative surgery, patients presenting with synchronous metastases were also excluded. A total of 113 patients met all inclusion criteria and were included in the final analysis. The study protocol was approved by the institutional ethics committee of our university hospital (approval number 2025-4044-BO-D, 01 December 2025).

### Data collection and statistical analysis

Patient characteristics, pathological findings, laboratory values, and imaging data were retrieved from the institutional hospital information system. Preoperative imaging studies were independently reviewed by two orthopaedic oncologists to assess tumour proximity to major vessels and to classify each case according to the Fujiwara system [18]:

- Type I: distance > 5 mm
- Type II: ≤ 5 mm and > 0 mm
- Type III: attached to the tumour ≤ 180° contact
- Type IV: vascular encasement/infiltration requiring resection

Interobserver agreement was excellent (κ = 0.92). Survival data were obtained from the regional cancer registry. All surgeries were performed by specialised sarcoma surgeons, and pathology was reviewed by dedicated sarcoma pathologists. Indications for radiotherapy were determined in a multidisciplinary tumour board for high-grade sarcomas; in selected cases treatment was omitted due to wound-healing complications.

Statistical analyses were performed using SPSS software, version 30.0 (IBM Corp., Armonk, NY, USA). All patients fulfilling the inclusion criteria and without exclusion criteria were included in the analysis. Missing data were excluded from individual analyses and not imputed. Accordingly, missing data are reported for each variable where applicable. Categorical variables were compared using Fisher’s exact test. As continuous variables were not normally distributed, non-parametric tests were applied (Kruskal–Wallis test for comparisons among more than two groups). Data are presented as median values with interquartile ranges (IQR).

OS was defined as the time from surgery to death from any cause or last follow-up. Median OS was estimated using the Kaplan–Meier method, and survival curves were compared using the log-rank test or, for continuous variables, by Cox regression analysis employing the Omnibus test. Multivariable Cox regression models were constructed to evaluate the independent prognostic impact of tumour–vessel proximity. Rather than relying on a purely significance-driven approach, a set of established prognostic confounders (age, tumour size, histologic grade, and resection status) was defined a priori and included in all models irrespective of univariate significance. In addition, selected clinically relevant variables (e.g., ASA score and tumour depth), as well as variables showing an association in univariable analyses, were considered for inclusion in the final multivariable models to improve clinical interpretability.

Given the limited number of outcome events (*n* = 47), the number of variables included in each model was carefully restricted to maintain model parsimony and reduce the risk of overfitting. Nevertheless, the events-per-variable ratio remains relatively low, and the results of the multivariable analyses should therefore be interpreted with appropriate caution. Because tumour–vessel proximity may correlate with tumour size or grade, multivariable findings should be interpreted cautiously. Formal collinearity diagnostics were not performed.

Tumour proximity to major vessels was incorporated in two ways: Model A used a dichotomised distance of > 1 cm vs. ≤1 cm, whereas Model B incorporated the Fujiwara classification as a categorical variable. Due to the small number of Type IV cases (*n* = 2), Fujiwara Types III and IV were pooled in multivariable modelling to improve statistical stability and precision of risk estimates.

To address multiple testing in the exploratory univariate Cox analyses, false discovery rate (FDR) adjustment according to the Benjamini–Hochberg procedure was applied, and adjusted p-values are reported. FDR adjustment was used solely to contextualise the univariate findings and was not used for variable selection in multivariable modelling. The proportional hazards assumption was verified for all variables included in the model. All statistical tests were two-sided, and a *p*-value < 0.05 was considered statistically significant.

## Results

### Baseline characteristics

The study cohort comprised 62 men and 51 women with a median age of 63 years (IQR, 51–75). During follow-up, 47 deaths occurred (all-cause mortality). Fifty-four patients were younger than 65 years, and 59 were aged 65 years or older. There was no significant age difference between sexes (*p* = 0.63). Median follow-up was 84 months (IQR, 48–120). Baseline characteristics are summarised in Tables 1 and 2. Missing data are reported where applicable.

**Table 1:** – Nominal baseline characteristics with respective median survivals

	number (*n* = 113)	Postoperative survival in months (95% CI)	*p* - value
Patient			
Male	62 (55%)	124 (92–156)	0.56
Female	51 (45%)	85 (67–103)	
Age < 65 years	54 (48%)	167 (54–280)	0.008
Age ≥ 65 years	59 (52%)	79 (41–117)	
ASA 1	7 (6%)	not reached (12-mo OS 86%; 95% CI 48–100%)	0.006
ASA 2	70 (62%)	124 (92–156)	
ASA 3	33 (29%)	44 (33–55)	
Missing	3 (3%)		
No other cancer	81 (72%)	107 (n/a)	0.70
History of other cancer	27 (24%)	85 (51–119)	
Missing	5 (4%)		
Married	50 (44%)	124 (31–217)	0.90
Not married	32 (28%)	85 (49–121)	
Missing	31 (28%)		
Tumour			
Pleomorphic sarcoma	43 (38%)	73 (27–119)	0.42
Liposarcoma	16 (14%)	167 (0–345)	
Myxofibrosarcoma	24 (21%)	124 (42–206)	
Other sarcoma	30 (27%)	87 (45–129)	
T1	16 (14%)	87 (43–131)	0.22
T2	50 (44%)	107 (n/a)	
T3	14 (12%)	41 (0–86)	
T4	15 (13%)	73 (n/a)	
Missing	18 (17%)		
Upper limb	23 (20%)	107 (21–193)	0.051
Lower limb	74 (66%)	85 (48–122)	
Trunk	16 (14%)	not reached (12-mo OS 94%; 95% CI 70–100%)	
Superficial	15 (13%)	not reached (12-mo OS 100%; 95% CI 78–100%)	0.018
Deep	79 (70%)	73 (37–109)	
Missing	19 (17%)		
> 1 cm to major vessels	45 (40%)	107 (n/a)	0.003
< 1 cm to major vessels	35 (31%)	42 (32–52)	
Missing	33 (29%)		
Fujiwara Type 1	47 (42%)	107 (n/a)	< 0.001
Fujiwara Type 2	15 (13%)	41 (0–146)	
Fujiwara Type 3	16 (14%)	44 (33–55)	
Fujiwara Type 4	2 (2%)	15 (n/a)	
Missing	33 (29%)		
Fujiwara Type 1	47 (42%)	107 (n/a)	< 0.001
Fujiwara Type 2	15 (13%)	41 (0–146)	
Fujiwara Type 3 + 4	18 (16%)	42 (21–63)	
Missing	33 (29%)		
Grading G2	48 (42%)	not reached (12-mo OS 98%; 95% CI 89–100%)	< 0.001
Grading G3	65 (58%)	44 (14–74)	
Treatment			
Operation 2004–2010	28	87 (26–148)	0.177
Operation 2011–2016	46	79 (16–142)	
Operation 2017–2020	39	not reached (12-mo OS 97%; 95% CI 87–100%)	
Planned resection	63 (56%)	85 (25–145)	0.45
“Whoops” resection	46 (41%)	not reached (12-mo OS 89%; 95% CI 76–96%)	
Missing	4 (3%)		
Resection in Centre	64 (57%)	107 (21–193)	0.94
Prior external resection	44 (39%)	87 (54–120)	
Missing	5 (4%)		
R0	58 (51%)	79 (28–130)	0.31
R1	35 (31%)	not reached (12-mo OS 89%; 95% CI 73–97%)	
R2	8 (7%)	33 (15–51)	
Rx	7 (6%)	not reached (12-mo OS 86%; 95% CI 42–100%)	
Missing	5 (5%)		
No radiotherapy	24 (21%)	85 (n/a)	0.81
Radiotherapy	84 (74%)	107 (65–149)	
Missing	5 (5%)		
No chemotherapy	98 (87%)	167 (61–273)	0.002
Chemotherapy	11 (10%)	22 (13–31)	
Missing	4 (3%)		
Outcome			
No local recurrence	56 (50%)	not reached (12-mo OS 96%; 95% CI 88–100%)	< 0.001
Local recurrence	51 (45%)	42 (32–52)	
Missing	6 (5%)		
No late metastases	65 (58%)	not reached (12-mo OS 94%; 95% CI 85–98%)	< 0.001
Late metastases	42 (37%)	36 (27–45)	
Missing	6 (5%)		

*p*-value of Kaplan-Meier analysis with Log-rank-test, *n/a* not applicable, *CI* Confidence interval, for groups where median survival was not reached, fixed-time survival at 12 months is provided

**Table 2:** – Metric baseline characteristics with respective median survivals

	Median	Inter Quartile Range	Hazard Ratio (95% CI)	*p*-value	FDR-adjusted *p*-value
Patient and tumour					
Age in years	63	51–75	1.026 (1.004–1.048)	0.020	0.105
BMI in kg/cm^2^	27.4	24.7–31.3	1.023 (0.976–1.073)	0.35	0.41
Maximal tumour diameter on MRI in cm	9	5.6–13.5	1.023 (0.978–1.070)	0.32	0.41
Treatment and outcome					
Time from initial symptoms to diagnosis in months	4.0	2.0–6.0	0.914 (0.839–0.996)	0.040	0.070
Distance from place of living to sarcoma centre in km	74.0	33.5–110.5	0.999 (0.992–1.005)	0.70	0.70
Time to recurrence in months (*n* = 51)	12	7.5–44.3	0.965 (0.934–0.996)	0.030	0.105
Time to late metastases in months (*n* = 42)	15	8–24	0.970 (0.944–0.997)	0.032	0.070

*p*-values of Cox regression with omnibus-test of postoperative survival*, FDR* False Discovery Rate

### Postoperative survival analysis

Median OS was 107 months (95% CI, 67–147). Survival did not differ by sex, tumour size, T stage, histologic subtype or tumour location (all *p* > 0.05; Table 1). Patients ≥ 65 years showed significantly reduced survival compared with younger patients (79 vs. 167 months; *p* = 0.008).

**Fig. 1:**
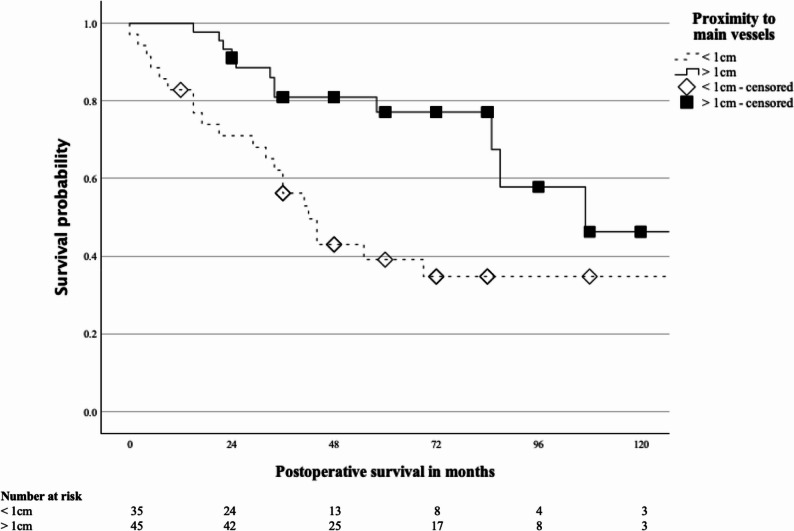
– Kaplan-Meier curve of postoperative survival of patients with soft tissue sarcoma depending on the distance to main vessels

A significant association was found between tumour proximity to major vessels and OS. Patients with a vessel distance greater than 1 cm had a median OS of 107 months (CI not estimable), compared with 42 months (95% CI, 32–52; *p* = 0.003) for those with a distance ≤ 1 cm (Fig. 1).

**Fig. 2:**
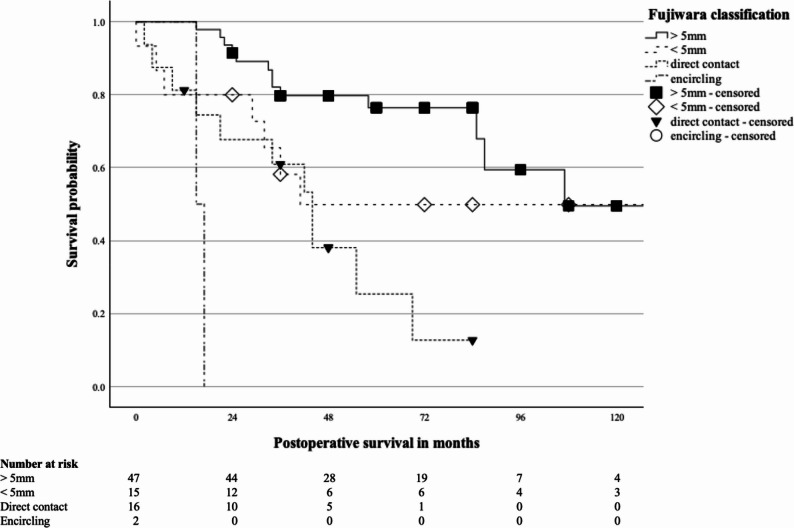
Kaplan-Meier curve of postoperative survival of patients with soft tissue sarcoma depending on Fujiwara classification

The Fujiwara classification showed significant differences in survival (*p* < 0.001; Fig. 2). Because only two patients were classified as Type IV, survival estimates for this subgroup were imprecise and confidence intervals were not reliably estimable; therefore, Types III and IV were pooled for multivariable analysis.

Higher histologic grade and deeper tumour location were also associated with decreased OS (*p* < 0.001 and *p* = 0.018, respectively).

Regarding treatment modalities, year of operation, primary surgery outside the centre, prior whoops procedure, R status, and radiotherapy showed no significant association with OS (*p* = 0.177, 0.45, 0.94, 0.31, and 0.81, respectively). In contrast, chemotherapy use was associated with reduced survival (*p* = 0.002), likely reflecting indication bias, as systemic therapy was preferentially administered to patients with high-risk disease. As expected, both local recurrence and late distant metastasis were strongly associated with poorer OS (each *p* < 0.001).

After FDR adjustment according to Benjamini–Hochberg, none of the metric variables—including age, BMI, maximal tumour diameter, time to treatment, distance to the treating centre, time to local recurrence, or time to late metastasis—remained statistically significant in the univariate Cox regression analysis (FDR-adjusted *p* = 0.105, 0.41, 0.41, 0.070, 0.70, 0.105, and 0.070, respectively; Table 2).

### Multivariable survival analysis

In multivariable analysis (Table 3), proximity ≤ 1 cm remained independently associated with impaired OS (Model A: HR 3.04; *p* = 0.006). In Model B, pooled Fujiwara types III/IV were independently associated with worse survival (HR 3.62; *p* = 0.005), alongside age ≥ 65 years and R2 resection.

**Table 3:** – Multivariable Cox regression analysis of overall survival including predefined confounders and tumour–vessel proximity

Model A – Proximity to main vessels assessed as +/- 1 cm		
Parameter	*P* value	Hazard Ratio (95% CI)
Age groups		
(< 65 years vs. >=65 years)	0.007	3.42 (1.39–8.40)
ASA Score 1	0.48	
ASA Score 2	0.77	0.78 (0.15–4.02)
ASA Score 3	0.78	1.28 (0.22–7.36)
Superficial vs. deep location	0.089	6.15 (0.76–49.81)
Grading (2 vs. 3)	0.050	2.412 (1.001–5.812)
Proximity to main vessels (< 1 cm vs. >1 cm)	0.006	3.04 (1.38–6.67)
R0	0.019	
R1	0.150	1.83 (0.80–4.16)
R2	0.002	9.67 (2.24–41.70)
Rx	0.75	0.70 (0.08–6.30)
Maximal tumour diameter in cm	0.92	0.966 (0.927–1.070)

Model B – Proximity to main vessels assessed by Fujiwara Scoring		
Parameter	*P* value	Hazard Ratio (95% CI)
Age groups		
(< 65 years vs. >=65 years)	0.015	3.07 (1.25–7.58)
ASA Score 1	0.43	
ASA Score 2	0.71	0.72 (0.13–3.96)
ASA Score 3	0.82	1.24 (0.19–8.08)
Superficial vs. deep location	0.071	6.79 (0.85–54.38)
Grading (2 vs. 3)	0.130	2.03 (0.81–5.07)
Fujiwara Type 1	0.017	
Fujiwara Type 2	0.119	2.29 (0.81–6.48)
Fujiwara Type 3/4	0.005	3.62 (1.47–8.94)
R0	0.042	
R1	0.191	1.75 (0.76–4.05)
R2	0.007	7.32 (1.70–31.47)
Rx	0.62	0.57 (0.06–5.33)
Maximal tumour diameter in cm	0.68	0.984 (0.914–1.061)

*p*-values of Cox regression with omnibus-test, *CI* Confidence Interval, *ASA* Score 1 as reference category for ASA Score, Fujiwara Type I as reference category for Fujiwara scoring; R0 as reference category for resection margins

### Correlation of outcome parameters with the Fujiwara classification

To further explore the mechanisms underlying the strong prognostic effect of the Fujiwara classification on postoperative survival, we examined its association with key postoperative outcome parameters. As shown in Table 4, the only significant correlation was observed for local recurrence (*p* = 0.011). This association may partially explain the observed survival differences; however, formal mediation analysis was not performed, and causal inference cannot be established.

**Table 4:** Correlation of Fujiwara types with outcome parameters

	Type 1	Type 2	Type 3	Type 4	p
No recurrence	28	9	3	0	0.011
Recurrence	18	4	12	1	
Time to recurrence (months)	30	11	6	-	0.100
	(12 – 64)	(3 – 11)	(3 – 28)		
No metachronous metastases	28	9	7	0	0.194
Metachronous metastases	16	5	9	2	
Time to metastases	14	19	16	4	0.189
(months)	(9 – 24)	(10 – 37)	(10 – 23)	(2 – 4)	

absolute numbers for nominal data; median with inter-quartile-range for metric data; *p*-values of Fisher’s exact test for nominal data and Kruskal-Wallis-Test for metric data

## Discussion

In this single-centre cohort study, we found that tumour proximity to major vessels was strongly associated with postoperative overall survival in patients undergoing curative-intent resection of deep soft tissue sarcomas. Importantly, the effect manifests in two complementary ways: when proximity was modelled as a dichotomised metric (> 1 cm vs. ≤ 1 cm; Model A), proximity remained an independent prognostic factor alongside patient age and resection status; when tumour–vessel relationship was instead captured by the Fujiwara classification (Model B), advanced Fujiwara types (III/IV) retained independent prognostic significance in addition to age and resection status. This pattern suggests that the Fujiwara classification provides complementary anatomical risk information rather than subsuming conventional prognostic factors.

### Mechanisms

Exploratory analyses suggest that the adverse prognostic effect of advanced Fujiwara types may partly be mediated by increased local recurrence rates. However, formal mediation analysis was not performed and causal inference cannot be established. Previous studies similarly reported higher recurrence rates and greater surgical complexity in tumours with advanced vascular contact [18–21]. Recent contemporary series continue to confirm established prognostic determinants in soft tissue sarcoma, including age, tumour size, and histologic grade [22, 23]. Moreover, pathological studies have highlighted vascular invasion as an adverse factor associated with disease progression, further supporting the biological relevance of tumour–vessel interactions [24]. These findings contextualise our results within current prognostic literature and suggest that anatomical proximity metrics may complement rather than replace established risk factors.

### Role of vessel resection/reconstruction

Vascular resection was performed in cases of radiologic encasement or clear intraoperative evidence of vessel infiltration, corresponding to Fujiwara Type IV. Surgical decisions were made by the multidisciplinary sarcoma team based on oncologic resectability and vascular involvement.

### Comparison of proximity metrics

The divergent multivariate results between Model A and Model B highlight a practical implication: a dichotomised distance threshold is a useful, simple predictor that retains independent association with outcome when adjusting for traditional factors; however, the Fujiwara classification—by incorporating circumferential contact and implied invasiveness— may offer more granular anatomical risk stratification within this dataset. This suggests that preoperative planning and prognostication may benefit from considering both simple metrics for broad triage and the more nuanced Fujiwara grading for detailed surgical strategy and patient counselling [10, 18].

### Clinical implications

For surgeons and multidisciplinary teams, the Fujiwara classification provides actionable preoperative information. However, because vessel resection may not fully abrogate elevated local recurrence risk—especially where neurovascular preservation limits resection width—discussion with patients should explicitly balance oncologic aims and functional outcomes.

### Strengths and limitations

This study benefits from several methodological strengths. The single-centre design ensured consistent preoperative MRI protocols, uniform application of the Fujiwara classification, and standardised surgical management.

Several limitations should be acknowledged. The relatively high local recurrence rate compared to contemporary series (typically ~ 20%) likely reflects the high-risk composition of our historical cohort. Moreover, the study was conducted at a specialised tertiary sarcoma referral centre, which may limit generalisability to non-specialised institutions with differing imaging protocols, surgical expertise, and case complexity. Only two patients were classified as Fujiwara Type IV; therefore, Types III and IV were pooled in multivariable analysis to improve statistical precision. Given 47 outcome events, residual overfitting cannot be fully excluded despite restriction of model complexity. The relatively low events-per-variable ratio represents a limitation of the present analysis and may affect the stability of the estimated hazard ratios. Fujiwara grading depends on MRI quality and reader expertise, which may affect reproducibility across institutions. The long inclusion period raises the possibility of temporal confounding, although year of surgery showed no association with outcomes in this cohort. While local recurrence appeared to mediate the adverse prognostic effect of vessel proximity, causal inference cannot be made without prospective studies. Finally, the absence of external validation in an independent multicentre cohort limits the generalisability of our findings, and prospective external validation is required before routine incorporation of tumour–vessel proximity metrics into prognostic algorithms.

## Conclusions

Tumour-vessel proximity is associated with overall survival and may provide additional prognostic information in high-grade STS. Both a simple distance threshold and the Fujiwara classification remained independently related to overall survival after adjustment for established risk factors. The association between advanced Fujiwara types and impaired survival appears partly linked to higher local recurrence rates. Prospective multicentre validation is required.

## Data Availability

The data presented in this study are not publicly available but available on request from the corresponding author. The data are not publicly available due to privacy and ethical restrictions.

## Abbreviations

ASA: American Society of Anesthesiologists
CI: Confidence Interval
FDR: False Discovery Rate
IQR: Inter Quartile Range
MRI: Magnet Resonance Imaging
OS: Overall Survival
STS: Soft Tissue Sarcoma
WHO: World Health Organisation
